# Mr. Huggan, on Cold Water in Convulsions

**Published:** 1806-01

**Authors:** A. Huggan

**Affiliations:** Wallingford


### To the Editors of the Medical and Phyſical Journal.

GENTLEMEN,

MR. SIMMONS having observed, in the treatment of his case of puerperal convulsions, (see your last Number p. 445) that "cold water was dashed upon the face of the patient, without abating the severity of the paroxysms," I am induced to send you another of the same kind, in which, cold water was so applied as to have had the effect of preventing their return, and which I shall briefly relate.

Mrs. H. ætat. 20, a well-made little woman, was on Sunday, the 2d of January 1803, delivered of a dead child, in the eighth month of her first pregnancy. The labour, which was soon over, was conducted by a midwife, without, as far as I could learn, a single untoward occurrence; the lochial discharge was scanty, and of a pale colour; but as she was as well in other respects as could have been expected, that was not regarded as a matter of any consequence.

On the Sunday evening following (9th), she complained of a head-ach, and, as her husband expressed it, "had an odd look, and did not seem to care for any thing." She grew daily worse, and on Wednesday was seized with convulsions, which continued till Saturday morning, with intervals sometimes of about three hours, but oftener, of an hour and a half only.

The medical treatment, before I first saw her, which was on Friday, the 14th, a little past noon, consisted chiefly in cordials, with opiates, æther, the warm bath, a blister between her shoulders, and sinapisms to her feet. At this time she was perfectly comatose, pupils dilated, and insensible to the usual stimuli; pulse varying from 150 to 160, and very weak; breathing slow and regular, which was the only favourable circumstance in the case of our patient.

I directed fifteen gr. of musk to be given her, every two hours; six dr. of laudanum to be rubbed into the inside of her thighs; a large blister to be put to the abdomen; and her hair to be cut off, for the purpose of making any application to the head that might be judged necessary; cold water was dashed upon her face during the fits, but without producing any effect at all. She took two or three doses of the musk regularly, and I thought, with some some good effect, as the paroxysms seemed not quite so severe, and the intervals between them longer; but soon after midnight, they returned with their former violence and frequency. We now found it almost impossible also to give her any medicine, as nearly the whole of it was lost each time we made the trial, so that we now began seriously to despair of our patient's life.

Having observed, that always before the fits came on there was a considerable flushing of the face with an increase of heat, particularly about the head, I thought that if this morbid heat could be carried off, before the commencement of the convulsive part of the paroxysm, this latter might be prevented. Accordingly, about five o'clock in the morning, (Saturday) the period at which a fit was expected, when the face began to be flushed, &c. cold water with salt dissolving in it, was applied freely to her head, neck, and shoulders, so that the heat was carried off before it rose to the degree that had before excited the convulsions, and which, by this means, were prevented; or, to speak in the appropriate language of Darwin, the catenation of motions which constituted the paroxysm was thus dissevered. Though we succeeded this time in preventing the convulsive part of the fit; at half past six, the period at which it would have come on again, there were the same appearances, viz. increase of heat, and the other signs of its approach; cold water was therefore applied again, in the same way, and with the same good effect. We also observed the same, though not so obviously, about eight o'clock; but after this the fits did not return, though I was told by the attendants that there was a slight flushing of the face some time in the forenoon, but that they had applied the cold water, and it soon disappeared.

Before I left her, I desired the musk to be continued; but after a few trials, it was found to disagree with her; and though the dose was lessened, it still had the same effect, it was therefore laid aside altogether.—The after medical treatment was very simple, and but slightly stimulant, with an opiate at bed time, gradually increased from fifteen to thirty drops of laudanum, and a dose of castor oil occasionally, as we placed our chief dependance upon wine regularly and *judiciously* given her. In no instance of disease that I have seen, was Dr. Darwin's "Golden Rule" in the exhibition of wine, more necessary than in the case of Mrs. H. as a very small quantity beyond what was proper, caused her to be restless, face

 flushed flushed, and the pulsation to be hurried and irregular, so that no small degree of caution was found to be necessary in giving it, particularly in the first stage of her recovery.

About three days after the fits had ceased, Mrs. H's left eye was observed to be somewhat inflamed; and without any other appearance of disease, the cornea became opaque, and she gradually lost the sight of it entirely.

Her recovery upon the whole, though it was not retarded by any other occurrence, was very slow; her pulse for a long time continued weak and quick, and with an evident undulating motion, as noticed in a similar case, by Mr. Christie, (Med. & Phys. Journal, vol. 3, p. 452); even at the end of three weeks from the time I first visited her, it was still upwards of a hundred.

Nor was the return of her reason much less gradual, for on the same day (4th of February), though I had conversed with her for some time on the preceding Monday, she had not any recollection of me, more than if she had never seen me. She is now in perfect health, and has since borne a child at the full time.

The first thing worthy of notice in this case, was the length of time that intervened between delivery and the accession of the convulsions. Another was the loss of the sight of one of her eyes, which I am inclined to think, was connected with the disease (though I cannot explain in what way), as Mr. Hempted, a very respectable surgeon and accoucheur in this neighbourhood, told me of a similar occurrence in his practice.

There was a part of the treatment of this case, before I saw the patient, that I shall take the liberty of criticising. When I asked with what view the sinapisms were applied, I was told that it was necessary to excite an irritation in the system; this might, for any thing I know to the contrary, have been a good idea, but the means used to effect it were inadequate, as local pain and constitutional irritation do not mean the same thing. The patient's sufferings were aggravated by the sinapisms, even at the time that she was insensible of sound, motion, and every thing else; and she appeared to have been greatly relieved when they were taken off; the ulceration occasioned by them was afterwards so painful, as frequently to interrupt her sleep, or altogether to prevent it, and when it was so essential to her recovery. As a rubefacient, sinapisms may answer some good purpose, but as a veiscatory they can never be necessary, as they possess no advantage, in this respect, over the common blistering plaster, not to say any any thing of the consequent ulceration, which is always more painful and tedious in healing.

A word or two on the treatment of the patient, after I saw her, in which I shall be as brief as possible. The reason of applying a blister to the region of the uterus, might in *sound* be plausible enough, but it certainly was of no benefit; for, even after it had produced its *utmost* effect, the fits were observed to return with increased severity and frequency.

The opiate friction was a mere waste of time and expence, for if I had given the subject a moment's thought, I must have been convinced of the irritability of the whole system being so low, and the function of absorption consequently so languid, as not *then* to have been a likely channel through which either opium, or any thing else, could be conveyed into the system; and I was deterred by high authority, from using it in any other way, (see Dr. Hamilton's cases in the Annals of Medicine for 1801); though in Mr. Simmons's case it had a good effect.

Whatever benefit we ascribed in the first instance to the musk, it certainly had none afterwards, but seemed rather to have been hurtful, so that we thought proper to discontinue it.

But, to conclude, a case is mentioned by Dr. Denman,* in which cold water applied (as above in that of our patient) in the same stage of the paroxysm had the same effect.

In my communication in your last number, (page 542) you should have added at the end of the paragraph containing the case of Lieutenant B. that he died with all the symptoms of hydrophobia, though the event would readily be anticipated.

I am happy to observe, that all the reports that have of late been circulated, with a view of prejudicing the cow-pox inoculation, and the *numerous failures*, &c. that have been announced with so much *academic* pomp and pedantry, have not been able to shake the well grounded confidence which we, of this part of the country, have in its preventive efficacy; as such *failures* appear to have no better authority than that of caprice or *dotage*. As to the few well authenticated instances of the small-pox occurring in individuals who have had the cow-pox, the most that can be made of them amounts only to this, that they are deviations from general and established laws, in the human œconomy, and are most likely owing to the same kind

* Introduction to Midwifery, vol. 2, note at page 378.

kind of *constitutional peculiarity* which in more than a proportionate number of cases, has caused not only the variolous inoculation, even where it excited the disease, but also the casual small-pox in its malignant form, to *fail* in preserving the constitution from a second attack of it. In proof of this, see the last four or five Numbers of the Medical Journal.

Some time last February, the poor of a neighbouring parish were inoculated with the cow-pox; a great many of whom did not take the disease, and therefore, as I understood, were inoculated again from those who had it. Ten or twelve of those who had been inoculated the second time, and who were supposed *now* to have taken the disease, and had partly gone through it, were shewn to me; when I found that at least three of this number had not the appearances of the genuine cow-pox, nor any mark that at all resembles it: so that it is obvious, that the ignorance or inattention of inoculators, is still a fertile source of many supposed failures.

I am, &c.

*Wallingford, Dec.* 13, 1805. A. HUGGAN.

